# Advanced Machine Learning Modeling Approach for Prediction of Compressive Strength of FRP Confined Concrete Using Multiphysics Genetic Expression Programming

**DOI:** 10.3390/polym14091789

**Published:** 2022-04-27

**Authors:** Israr Ilyas, Adeel Zafar, Muhammad Talal Afzal, Muhammad Faisal Javed, Raid Alrowais, Fadi Althoey, Abdeliazim Mustafa Mohamed, Abdullah Mohamed, Nikolai Ivanovich Vatin

**Affiliations:** 1 University of Science and Technology (NUST), Sector H-12, Islamabad 44000, Pakistan; israr.awaan@gmail.com (I.I.); adeel.zafar@mce.nust.edu.pk (A.Z.); muhammad.talal.afzal@gmail.com (M.T.A.); 2 Punjab Irrigation Department, Government of Punjab, Old Anarkali Road, Lahore 54000, Pakistan; 3 Department of Civil Engineering, Abbottabad Campus, COMSATS University Islamabad, Abbottabad 22060, Pakistan; 4 Department of Civil Engineering, Jouf University, Sakaka 72388, Saudi Arabia; rnalrowais@ju.edu.sa; 5 Department of Civil Engineering, College of Engineering, Najran University, Najran 1988, Saudi Arabia; fmalthoey@nu.edu.sa; 6 Department of Civil Engineering, College of Engineering, Prince Sattam Bin Abdulaziz University, Alkharj 16273, Saudi Arabia; a.bilal@psau.edu.sa; 7 Building and Construction Technology Department, Bayan College of Science and Technology, Khartoum 210, Sudan; 8 Research Centre, Future University in Egypt, New Cairo 11835, Egypt; mohamed.a@fue.edu.eg; 9 Peter the Great St. Petersburg Polytechnic University, 195291 St. Petersburg, Russia; vatin@mail.ru

**Keywords:** CFRP, modelling, machine learning, GEP, strength model, confinement, gene programming, artificial intelligence

## Abstract

The purpose of this article is to demonstrate the potential of gene expression programming (GEP) in anticipating the compressive strength of circular CFRP confined concrete columns. A new GEP model has been developed based on a credible and extensive database of 828 data points to date. Numerous analyses were carried out to evaluate and validate the presented model by comparing them with those presented previously by different researchers along with external validation comparison. In comparison to other artificial intelligence (AI) techniques, such as Artificial Neural Networks (ANN) and the adaptive neuro-fuzzy interface system (ANFIS), only GEP has the capability and robustness to provide output in the form of a simple mathematical relationship that is easy to use. The developed GEP model is also compared with linear and nonlinear regression models to evaluate the performance. Afterwards, a detailed parametric and sensitivity analysis confirms the generalized nature of the newly established model. Sensitivity analysis results indicate the performance of the model by evaluating the relative contribution of explanatory variables involved in development. Moreover, the Taylor diagram is also established to visualize how the proposed model outperformed other existing models in terms of accuracy, efficiency, and being closer to the target. Lastly, the criteria of external validation were also fulfilled by the GEP model much better than other conventional models. These findings show that the presented model effectively forecasts the confined strength of circular concrete columns significantly better than the previously established conventional regression-based models.

## 1. Introduction

Recently, an upsurge has been recorded in using fiber-reinforced polymer (FRP) as an exterior enclosure for structural components when the interior reinforcements become ineffective. The retrofitting and rehabilitation of buildings and bridges affected by seismic events can also benefit from using advanced composite materials like FRP and steel jacketing as exterior confinement [1,2,3,4]. In addition to being lightweight and strong, FRPs are also weather and electromagnetic resistant, and easy to substitute on structural components that have been damaged. FRP composites are being used to rehabilitate and upgrade structures affected by earthquakes, thus enhancing their resilience and stability. Fiber-reinforced concrete structures have been the subject of several publications, including (but not limited to) [5,6,7,8,9,10]. Several empirical models have been established so far [11,12,13] to anticipate the response of FRP-wrapped concrete at axial loads. Moreover, based on explanatory variables as the ultimate conditions for fiber reinforced concrete specimens, Fardis and Khalili in 1982 [14] evaluated the model proposed by Richart [15] and Newman [16]. These empiric models were developed using conventional regression techniques and also by considering limited (small numbers of data points) geometrical and mechanical properties of FRP-based confined concrete, which cannot ensure accuracy and applicability. Subsequently, the majority of models were gradually developed by employing a consistent set of variables over time, principally by incorporating both the strain ratio and the lateral confinement ratio in most cases, whereas the role of many significant contributing factors has not been investigated using a variety of modeling approaches. Sadeghian and Fam [17] analyzed a range of models, and chose the best fit using a regression model. Keshtegar et al. [18] employed modified standard harmony search methods for resolving complex nonlinear system modeling problems, whereas Keshtegar et al. [19] introduced a chaotic dynamical management approach that maintained the polynomial mapping nonlinearity while obtaining undisclosed model parameters using fresh and previous trials. As previously stated, previous investigations were constrained by the small datasets employed, which impeded their capacity to apply their findings to unknown data [20,21,22].

Mander et al. [12] developed empiric models for assessing the flexural strength and strain of columns directly constrained with fiber-reinforced polymer (FRP) by employing the energy balance technique. The energy balancing approach assumes that the energy needed to burst the transversal FRP isolation is equivalent to the increased strain energy of concrete confined with FRP. The FRP confinement model proposed by [22] were based on the strain capacity and dilation tendency of concrete, which were incorporated by ACI [23] in 2008 with subsequent variations. When prior models were developed in the 1990s, the ruptured strain of FRP was ignored in favor of the maximum tensile strain specified by the fabricator. In the last several years, investigations have shown that the true strain required to cause an FRP to rupture is much lower than previously anticipated [1,22].

Artificial intelligence (AI) approaches are being utilized to evaluate civil engineering phenomena worldwide [24]. For instance, researchers have employed a variety of AI approaches, including RS (response surface technique), ANFIS (adaptive neuro-fuzzy interface system), NN (neural network), RF (random forest), PSA (particle swarm algorithm), GA (genetic algorithm), GP (genetic programming), and GEP (gene expression programming) [25,26,27,28,29,30]; however, Naderpour [31] employed Artificial neural networks (ANN) to obtain the relationships within confined concrete through fiber-reinforced polymers. Elsanadedy et al. [32] again uses the confinement model based on the neural networks (NN) technique. Recently, many other studies have also evolved in an attempt to better cater to, and analyze the behavior of, confined concrete, either focusing on ultimate conditions or by using distinctive techniques such as regression, artificial neural networks (ANN), stepwise regression (SWR), or a support vector machine (SVM). Moreover, other types of approaches such as the Bayesian principle or algorithms such as whale optimization networks [33,34,35] have also been used. These studies employed small databases, which makes their approach a little less mature compared with studies that make used of a vast range of datasets. Although algorithms such as ANN, as well as ANFIS, are capable of recognizing and generalizing subtle differences, they are also sometimes efficiently utilized to tackle technical challenges [36]. Due to the presence of massive neurons in the hidden layer, this makes the link development among the source and target parameters difficult. Even though these approaches sometimes show a good association between outputs and inputs, they did not have an analytical expression that could be employed in practice. This is because of the intricate architecture of ANFIS and ANN algorithms, which hinders the widespread implementation of such approaches [37,38,39,40]. Various other researchers [41,42,43,44,45,46,47] also developed different mathematical models for the evaluation of multiple complex engineering problems.

Gene Expression Programming (GEP) is a desirable Multiphysics model, as it overlooks preceding established associations in the development of a model [48,49,50,51]. Subsequently, GEP, a variant of gene programming (GP), is used in structural engineering. GEP encodes a program on a fixed-length linear chromosome [52]. It can provide a simple analytical expression for forecasting the behavior that can be utilized in practice [53,54,55,56]. As per the authors’ knowledge, no study has been conducted that achieves a larger database in studies concerning CFRP and GEP. In the literature, there are some other models that have been identified based on neural networks, but they do not provide an equation. In contrast to neural networks such as ANN and ANFIS, GEP offers several advantages over these techniques. When talking about ANN algorithms, their worthlessness is important, due to their inability to provide a working relationship or framework that can be utilized in practice; however, the output in GEP is obtained in the form of an expression tree which is later decoded to get a simple mathematical relationship that is easy to use and can be applied to future predictions. Establishing frameworks is a special ability of GEP, which demonstrates its novelty by providing simple and reliable models [57], making this approach even more reliable in providing accurate predictions for the future, whereas all other techniques except genetic programming are only used as predictors. Considering these limitations, neural networks are categorized as black-box algorithms that hinder their application; therefore, GEP as an alternative to these techniques takes over other approaches to tackling engineering and complex issues. Cevik et al. [58] initially attempted to utilize GEP in his work to develop a model for strength enhancement. Later, Gandomi et al. [59] studied the complex interaction and behavior between FRP and concrete. In [60], a similar approach was used for axial compression of confined concrete. In [60] the author has generalized his study by incorporating distinctive natures of confinement wraps having different mechanical properties (CFRP, GFRP, and, AFRP), along with some tube encased specimens in the database, thus resulting in unaligned behavior. These models make their accuracy and reliability less efficient, and sometimes inaccurate, due to limited databases, which is an important aspect of modeling on machine learning. Moreover, a small number of data points make it difficult for AI approaches to efficiently distribute and randomize the sample datasets evenly in phases (i.e., training, testing, and validation). This uneven distribution, with respect to specific data sets, sometimes leads the problem towards overfitting, collinearity, or other similar issues that may arise when modeling.

To avoid these issues and present a model with more generalizability and reliability, experiments on 828 specimens conceived from previous research have been used in this study to develop a predictive model for the compressive strength of fiber reinforced concrete with an exterior confinement. This new model was chosen after an extensive assessment and review of the existing strength models in the database that had been set up. The generated model was validated using validated sets and external validation criteria. Moreover, a parametric analysis was conducted to analyze the influence of individual variables on the strength of concrete enclosed by FRP. The findings of this research will aid sustainable construction in its efforts to better interpret the structural performance of fiber-reinforced concrete.

The fundamental objective of this research is to develop a strength prediction model for FRP-confined concrete cylinders. Strength models have been studied before, but the data used was substantially smaller or limited. Correlation coefficients (R) and mean square errors (MSE), among other statistical indicators, were also employed to examine the experimental results of confined cylindrical specimens enclosed with FRPs. The theoretical models that were created based on conventional regression techniques for FRP-confined concrete were used to draw the comparison. As an additional authentication step, MLR and MNLR models were also developed and compared with the newly developed AI model; however, analysis of existing and newly developed GEP models shows that the proposed model for the CFRP-confined concrete members outperforms the previous ones.

## 2. Gene Expression Programming

**Figure 1:**
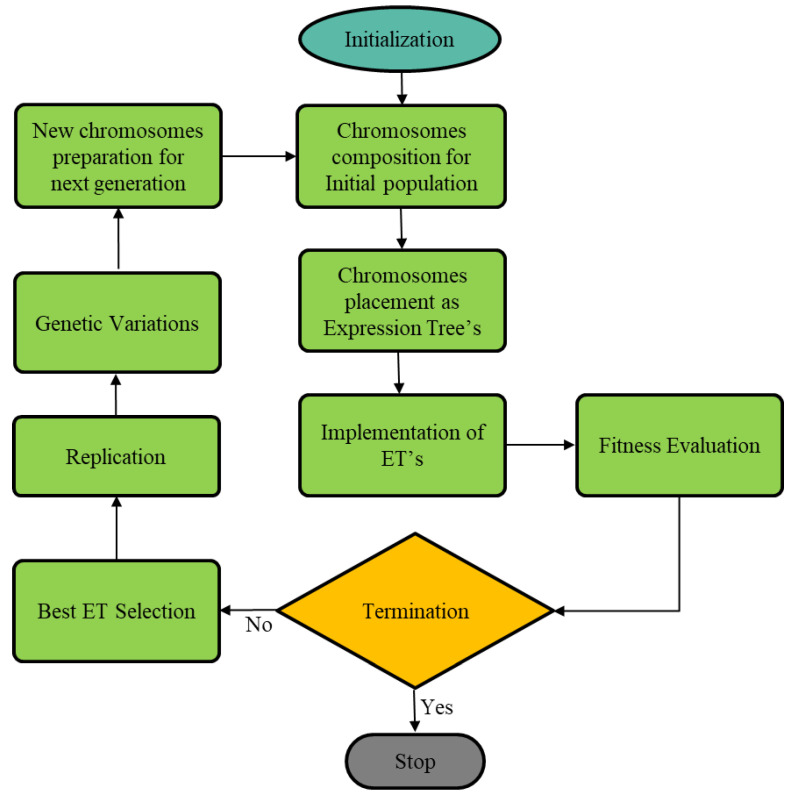
GEP Flow diagram.

It was Ferreira [61] who originally introduced the genotype branch of the evolutionary computational approach, which emphasizes the importance of biological evolution essentials to transform computer programs. As the chromosomes are linear, GEP can be thought of as an amalgamation of GA and GP, since it uses GP to encode and parse trees of various configurations. GEP uses fixed-length chromosomes composed of one or more genes to evolve computer programs. Functions, variables, and constants are the building blocks of symbols in GEP, which are referred to as basic components. Parameters and constants are known as terminals because they do not require any more information. Thus, a gene is formed by an organized set of symbols, then an organized set of genes forms a new chromosome. It is common in GEP systems to have between 4 to 20 symbols per gene, and for each chromosome to normally consist of one or several genes. For each GEP gene, there is a fixed-length structure composed of tiny subprograms with a head that mostly contains function sets and operators such as +, −, ×, ÷, and a tail that only contains variables and constants. For each task, a random set of fixed-length chromosomes is generated. After that, the solution to a particular issue is appraised. For many generations, the process repeats itself until the desired outcomes are achieved. Mutation, reproduction, and crossover are all utilized during iterations to manipulate organisms and corresponding populations. The workflow diagram for the proposed GEP algorithm is shown in Figure 1.

A major advantage of the Genomic-based Operationalized Programming technique is that genetic operators can be used at the chromosomal level to create biological variation without requiring a complex production process or resourceful ways to conduct genetic operations [61]. Due to its distinctive and multigenic characteristics, GEP transforms multi-subprogram systems in much simpler ways [62,63]. When it comes to solving complex issues, GEP uses Darwinian concepts of inherited traits, along with variability and adaptation.

However, when the objective is to achieve forecasting models using GEP, then the data sets need to be defined, and the major objective is to develop models connecting them without any previous assumptions. The Karva script is used to pick gene expression patterns and operators, which results in a random number of individuals (chromosomes) in the GEP process. Tree expressions are created from K expression in the genome because algorithms can assess and develop tree expressions much more smoothly than K expressions. An objective function is then used to categorize the individuals in the population. A greater percentage of the descendants of individuals with better fitness scores will be permitted to form parents in the coming generation. Members are then randomly mutated (recombination or crossover), transposed, or rearranged [49]. The process will continue to evolve towards convergence, but once the convergence conditions have been met and the fittest member has been picked up, the process could be terminated by specifying a different criterion, such as restricting the number of generations or the amount of time that has elapsed.

## 3. Research Methods

### Database Establishment and Division

**Figure 2:**
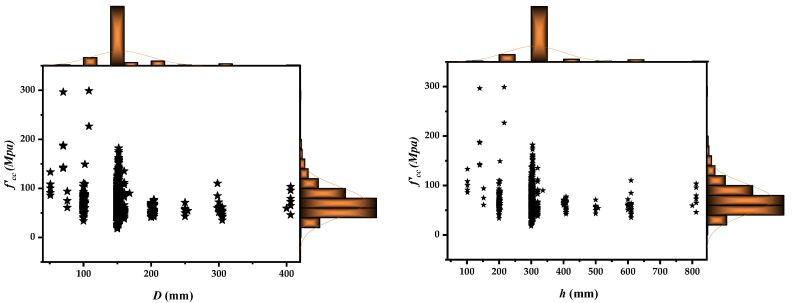

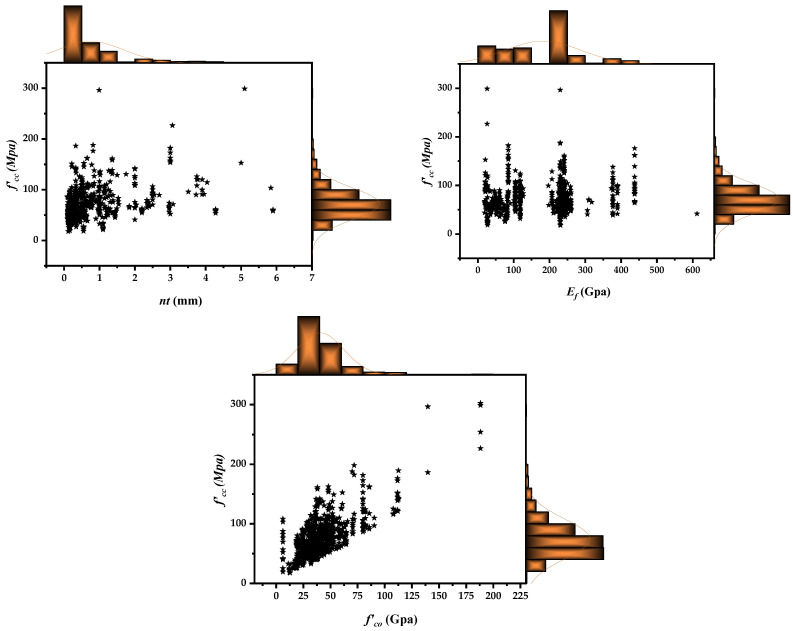
Marginal histograms for each predictor variable.

After a detailed assessment of the literature review, the database has been compiled. Incomplete information and details were excluded to maintain the adherence and accuracy of developed model datasets; however, the effect of mix design and other factors such as curing conditions were not taken into account. These factors do not directly affect the performance of FRP, rather, they affect the concrete strength. Thus, in the literature, there are multiple strength models on concrete that have been developed separately in the past by considering mix proportions and multiple other factors [64,65]. If these factors were considered in the confinement model, then it would be too complex to deal with and obtain a working relationship. Moreover, other confinement models in the literature also did not account for either mix design or other curing conditions, rather, they directly employed the unconfined concrete strength [35,66,67]. Around 830 CFRP confined concrete tested experimental results were compiled into a large database. To date, as per the authors’ knowledge, this is the maximum number of experimental results available in the literature. Thus, to make an effective model, the maximum numbers that could be employed were used in this study, in an effort to make the model more generalized and robust. Moreover, in the literature, researchers recommended that the proportion of the total number of data points compared with the number of explanatory variables (that were employed for training, validation, and testing sets) needs to be greater than 5 [68]. Furthermore, with more datapoints in the model development process, the probability of an efficient and robust model is increased; however, in light of the mentioned criteria, for the training phase, the evaluated model has a ratio of 116, whereas in the testing stage, the f’cc model has a ratio of 24.8. Moreover, various statistical metrics have been used to evaluate the robustness of the strength models of these databases. It has been observed that the elastic modulus of CFRP ranges between 10 and 612 (GPa), indicating there are two categories of FRPs, namely, those with a low and a slightly elevated elastic modulus, respectively. Additionally, the database has a range of values for concrete columns confined with FRPs that are up to 812 mm tall and have an axial confined compressive strength (f’cc) of 302.2 MPa. The marginal histogram presents the distribution of each explanatory variable as shown in Figure 2. As can be seen in Figure 2 the data is distributed all over its range for each explanatory variable. For diameter and height, the maximum values lie in the ranges of 400 mm & 800 mm respectively. Similarly thickness and elastic modulus parameters have also well versed distribution. However, these distributions play significant role in capturing over all behavior of model. Lastly the data for unconfined concrete is distributed proportionally along the response variable which also provide insight regarding relationship between response and explanatory variable.

**Figure 3:**
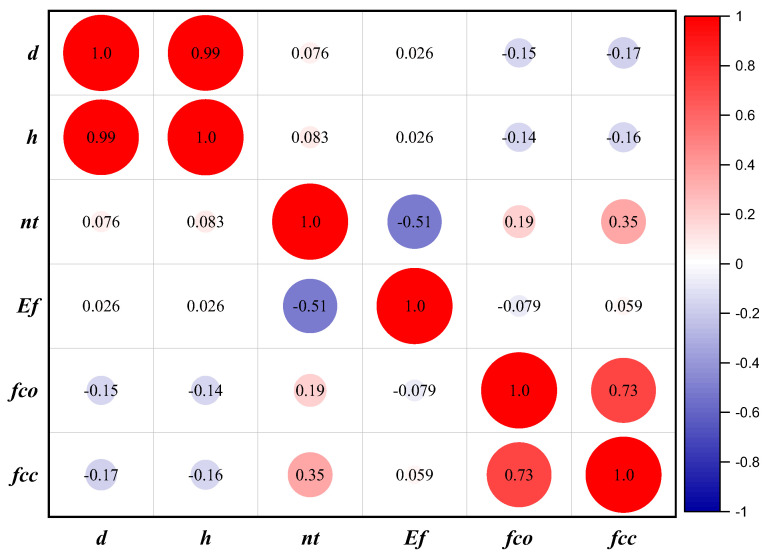
Correlation plot based on explanatory variables.

An issue that arises in implementing machine learning approaches is multicollinearity, which arises due to the dependency of process variables [69]. Increasing correlation strength and durability between variables can reduce the model’s performance. It is advised that the correlation (R) value between two input parameters needs to be less than 0.8 [70] to address the risk of collinearity. According to Figure 3, R is evaluated for all possible input variables. The table in Figure 3 indicates that R, either negative or positive, falls below the stated limit of 0.8, demonstrating that there would be no potential for collinearity during modeling.

GEP has a distinct edge in that it does not require or play a limited part in reprocessing input and output data, including data normalization. For modeling, GEP generates computer programs or chromosomes with functions that are relevant to the problem model and data distributions, allowing input and output variables to be readily inputted for modeling. Algorithmic and numerical computation approaches have an issue known as overfitting that makes it difficult to generalize their models. In this situation, the training set’s error is reduced to a very low number, but the deviation increases significantly when fresh data is introduced to the model. As part of the selection process for training models, validation datasets are categorized data sets. Other individuals must be tested on a validation dataset to locate the best generalization [71] to minimize overfitting in GP and its derivatives. Additional test data can be incorporated into models to enhance their generalizability and thereby improve the accuracy of predictions. The accessible data sets are therefore divided into three groups based on a random selection: (i) training subsets, (ii) validation/check subsets, and (iii) testing subsets. There have been various published assessments concerning the proportions of data used to build the model using artificial intelligence (AI), ranging from 50–70%, 15–25%, and 15–25%, respectively [72,73,74,75,76]. As part of the model selection criteria, a train set is employed to develop the initial model, whereas a validation set is used to assess estimation error, and a test set is utilized to evaluate the generalization of the model’s projected error. In this study, around 70% of data have been used for training, and the rest of the data, at around 30%, have been used for validation and testing the models individually.

## 4. Modeling Approach

### 4.1. GEP Modelling

A credible database has been used to establish five explanatory variables: *d* (mm), *h* (mm), *nt* (mm), *f_co_* (MPa), and *E* (GPa), to build a prediction model for the *f_cc_* (MPa) of confined concrete columns through a reliable database. Dependably, GeneXpro Tools 5.0 is employed in this work to develop the GEP model.

When using GEP models, the parameter settings might have an impact on the models’ reliability and generalizability. Consequently, several runs are therefore necessary to come up with a reliable and stable model. When it comes to configurations, they are being modified in each run by an iterative process, or they can be employed as sets of parameters, as previously suggested by researchers [49,74,76]. For this study, the model creation process has been governed by taking into account distinctive factors indicated in Table 1. It is important to remember that increasing the number of genes itself does not lead to a significant enhancement, but it might lead to overfitting [63,77]. As shown in Table 1, the optimal GEP model does not necessarily contain all of the arithmetic and logical operations that were used to demonstrate this.

To establish the GEP paradigm, we need to understand the structure of the genome, genes, and expression trees. The population’s number determines the length of the program. The chromosome is made up of genes that encode the subexpression trees. According to the intricacy of the forecasting model, 160 points were chosen as population numbers. Each term is determined by the number of genes and head-size, determining how difficult each term is and how many sub-ETs make up the model’s aggregate; hence, the model takes into account a population of 160, having 3 genes, and a head size of 8. Through genetic operators, chromosomes are susceptible to genetic changes. Mutation occurs when a randomly chosen element of the gene’s tail or head is swapped with a randomly generated element in the expression tree. The transposition is responsible for the sequences’ transposition inside chromosomes, specifically two major sequences, the insertion and root insertion sequences. The architecture framework for the proposed study has been employed to establish an adequate prediction model. Table 1 shows the synchronized hyper parameter configurations used to build the analytical expression.

The optimal GEP frameworks could be derived from assessing a fitness function following the training phase. A tree-like representation of the eventual GEP model can be depicted in the chromosomal mode. Here, the generated tree-shaped expression is simplified and turned into a functional representation to make it easier to utilize via hand computations using consecutive replacements of variables. Equation (1) shows the best GEP-based formulae for indirect determination of the CFRP confined concrete columns. The best GEP model can be determined by taking into account the preceding modeling challenges and issues during the preliminary phases of the GEP approach. As indicated by a fitness function, the aptitude of the model is the criteria for choosing the most suitable GEP model among those developed. It is important to keep in mind that developing an effective and precise model requires more than just one criterion. Consider that statistic regression modeling tends to be obtained by manipulating just a few prior solutions. As a result, for each of these models, the interconnections among the explanatory and response variables are not considered, but in GEP, an appropriate mathematical model is formed by suppressing multiple linear and nonlinear based baseline models, mostly introduced via genes and associated chromosomes.

*f_cc_* = A + B + C, (1)

$$\begin{array}{l} A=\frac{\left(\mathrm{nt}-15.269\right)\;*\;\left(-64.1539-\mathrm{Ef}\right)\;*\;\sqrt[{3}]{\mathrm{nt}}}{D},\\ B=\sqrt[{3}]{53.32*f_{co}^{2}*h-\frac{Ef}{nt}}\\ C=\sqrt[{3}]{f_{co}+\left(nt-0.00793\right)*\left(Ef+f_{co}\right)*2h}, \end{array}$$

### 4.2. Regression Models

Several statistical metrics are evaluated to forecast GEP accuracy and performance. *R^2^* is the coefficient of correlation, which is one of these. In fact, it cannot be enough to classify a model on its own because it does not forecast the outcome of a constant’s division or multiplication.

Regression models are initially compared with one another. Afterwards, the GEP model results are compared with the best MLR and MNLR results. Thus, after comparing the results, the multiple nonlinear regression (MNLR) model is preferred over the MLR model.

#### 4.2.1. Multiple Non-Linear Regression (MNLR)

Modeling the nonlinear relationship between two variables is a common practice in statistics. It is utilized instead because linear regression could not often yield accurate findings similar to nonlinear regression in certain cases. This happens primarily due to the fact that the data in linear regression is considered straight. The MNLR approach uses nonlinear relationships to collaborate with dependent and independent variables. MNLR models can appear mathematically as:

(2) $$Y=b+\beta_{1}\chi_{i}+\beta_{2}\chi_{j}+\beta_{3}{\chi_{i}}^{2}+\beta_{4}{\chi_{j}}^{2}+\ldots +\beta_{q}\chi_{i}\chi_{j}$$

where b corresponds to intercept, β denotes the slope of the line, and q is the number of instances. The relationship in Equation (2) is utilized to forecast the behavior of Y with respect to χ.

#### 4.2.2. Multiple Linear Regression (MLR)

MLR refers to a method in which the interaction among response and predictor variables has a straight-line behavior. When using linear regression, we are more concerned with the regression coefficient *(*β*)* that has the least impact on the model’s overall inaccuracy by dealing with the discrepancies.

(3) $$Y=b+\beta_{1}\chi_{j}+\beta_{2}\chi_{j}+\beta_{3}\chi_{j}+\ldots +\beta_{q}\chi_{j}$$

In Equation (3), Y expresses the forecasted confined compressive strength of CFRP concrete. The variable “χ” represents the explanatory variables that were used to develop the equation.

## 5. Results and Discussion

### 5.1. Model Performance and Evaluation

**Figure 4:**
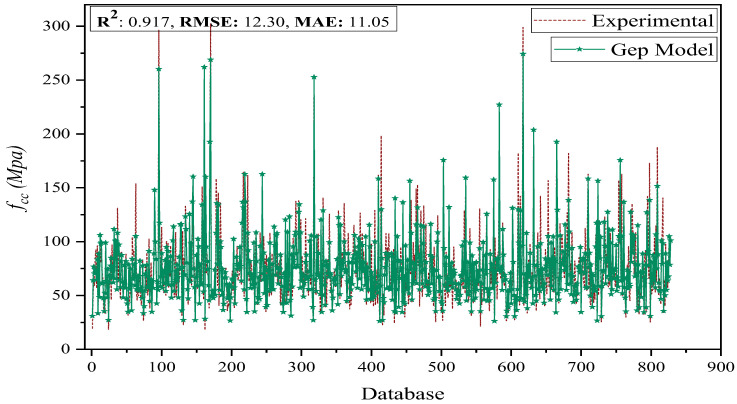
Forecasting via GEP against actual output.

It is necessary to undertake numerous supplemental processes before selecting a suitable model, and the efficacy and correctness of the model should be thoroughly assessed. Assessments based on statistic evaluation can facilitate this. It is recommended by [70] that models that provide a Pearson correlation (R) value > 0.8 will be considered adequate in terms of their ability to foresee future outcomes. Furthermore, the disadvantage of solely using R as just a benchmark for assessing the robustness of the model is that it is unresponsive to discrepancies in model predictions and actual values that are either additional or proportionate. As a result, additional factors must be taken into account; however, various metrics can be employed, such as root mean square error and mean absolute error. Disparities between expected and actual results can have a greater impact on such indexes, as they are more responsive to big, random deviations. RMSE is a metric for predictive performance that combines the intensities of forecasting flaws across different time periods into a single number. It assigns a relatively greater importance to extremely big discrepancies. In addition, MAE indicates the average size of the variance. The superior predictive performance will be achieved if RMSE and MAE values were relatively low. The formulae mentioned below can be used to figure out these indices:

(4) $$R=\frac{\sum_{i-1}^{n}\left(e_{i}-\bar{e}i\right)\left(m_{i-}\bar{m}i\;\right)}{\sqrt{\sum_{i-1}^{n}\left(e_{i}-\bar{e}i\right)²\sum_{i-1}^{n}(m_{i}-\bar{m}i)²}}$$

(5) $$\mathrm{RMSE}=\sqrt{\frac{\sum_{i-1}^{n}(e_{i}-m_{i})²}{n}}$$

(6) $$\mathrm{MAE}=\frac{\sum_{i-1}^{n}\left|e_{i}-m_{i}\right|}{n}$$

where *n* represents the size of samples and *e_i_* and *m_i_* are the averages of the measured and estimated output for the *i*th indices, respectively. It is recommended that the model’s efficacy is tested on a variety of datasets to ascertain if it overfits. The R, RMSE, and MAE evaluation metrics could be used to accomplish this. Data sets with low levels of overfitting in the R, MAE, and RMSE on the train and test sections support this claim. [49,71,74]; however, as a way to showcase the GEP models’ capabilities, the anticipated versus actual values are shown in Figure 4, and it demonstrates the behavior among developed AI models against experimental output. It can be seen that at almost every data point, the GEP model effectively forecasts the corresponding output with maximum accuracy. Statistical metrics of the proposed model against actual values depict that the model possesses high generality with low discrepancy values when incorporating such a huge database. Moreover, it is demonstrated through Figure 4 that GEP-based *f_cc_* models which have high R and moderate RMSE and MAE indices are capable of predicting the output with adequate precision; however, near R^2^, RMSE, and MAE, values on the data sets indicate that it has both superior forecasting and heuristic capabilities, coupled with the fact that overfitting is nullified.

**Figure 5:**
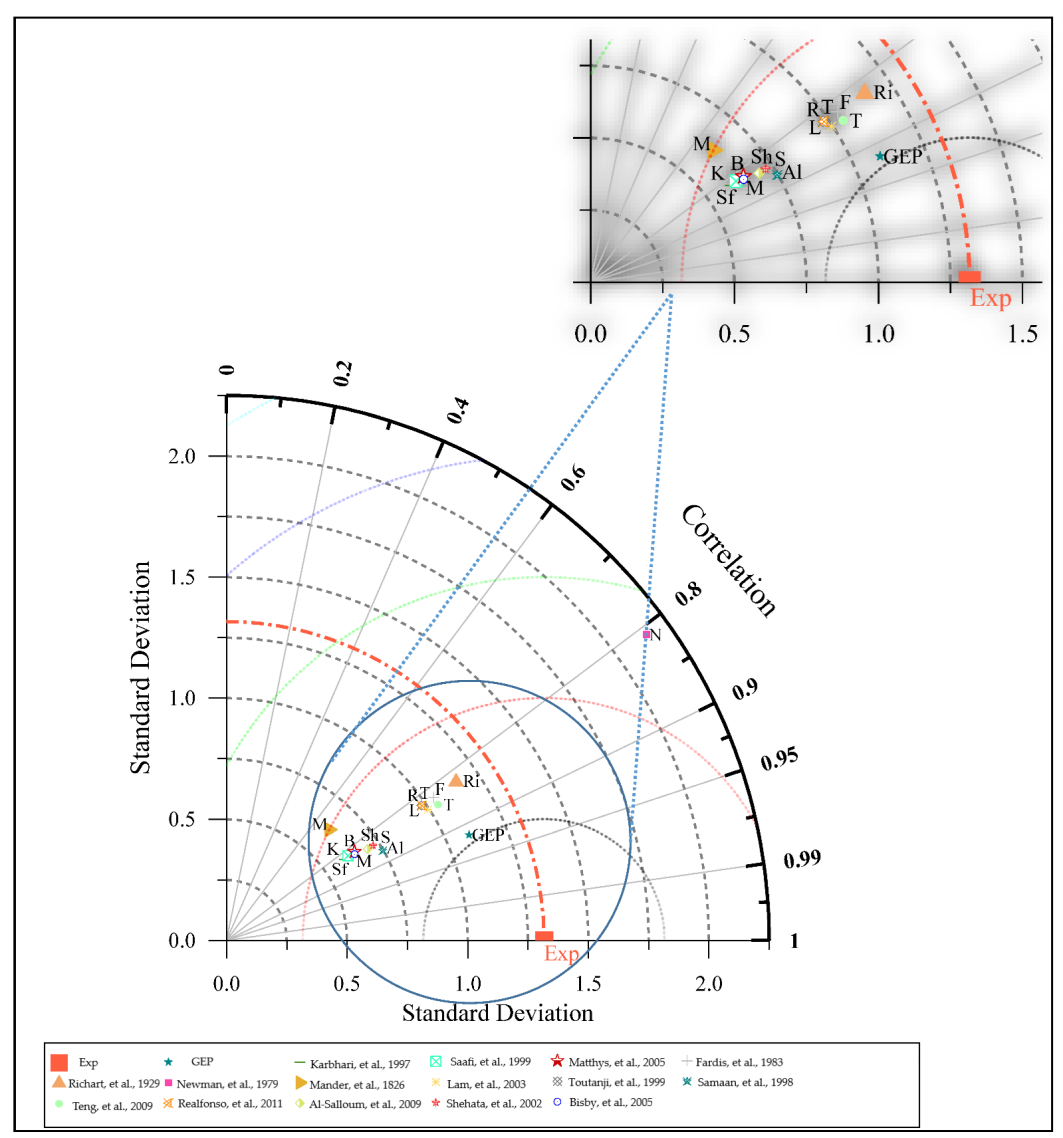
Taylor Diagram for performance evaluation of GEP and existing models [11,12,14,15,16,20,22,79,80,81,82,83,84,85,86].

A normalized Taylor diagram [78] in Figure 5 depicts the Pearson correlation coefficient, and the predicted standard deviation of presented the model for comparison purposes. The accuracy of simulated models is assessed by comparing their distance from the target point on a Taylor diagram. When it comes to forecasting outputs that needed to be close to experimental data, the GEP model surpassed the other models [79,80,81,82,83,84,85,86], as shown in Figure 5. Essentially, this is due to the large sample prediction capability and potential prevention of localized minimal occurrences, with an improved generalization performance of the GEP approach. It appears to be more effective in solving finite samples, complex, and function fitting issues compared with other machine learning (ML) techniques. Thus, for the calculation of the compressive strength of FRP-confined concrete, the newly developed ML model surpasses previous conventional approaches.

### 5.2. External Validation

Furthermore, the GEP model findings are being evaluated in empirical or other forecasting models in an attempt to further examine the model’s accuracy and validity. Several other metrics, in addition to R, MAE, and RMSE, may be taken into consideration in this context; however, for external validation, it is suggested that at least one regression line (*k* or *k’*) across the origin must have a gradient at approximately 1. [49,73,74]. Additionally, the *o* and *p* efficiency indices have to be smaller than 0.1.

Another indicator *R_m_* has also been developed by many scholars [49,73,74] to assess frameworks’ predictive power. The criterion is met if *R_m_* > 0.5. Both the squared coefficient of correlation (via the origin) among the forecasted and actual values (*R_o_^2^*), and the correlation among the actual and forecasted values (*R_o_’*^2^), must be near *R*^2^ and 1. As shown in Table 2, o*_i_* and *p_i_* indicate the observed and anticipated results for the *i*th yield, accordingly, and n represents the total number of observations taken. To illustrate the validity of the validation criteria, Table 2 shows the findings produced by various approaches (AI and Regression models) for *f_cc_* (MPa).

**Figure 6:**
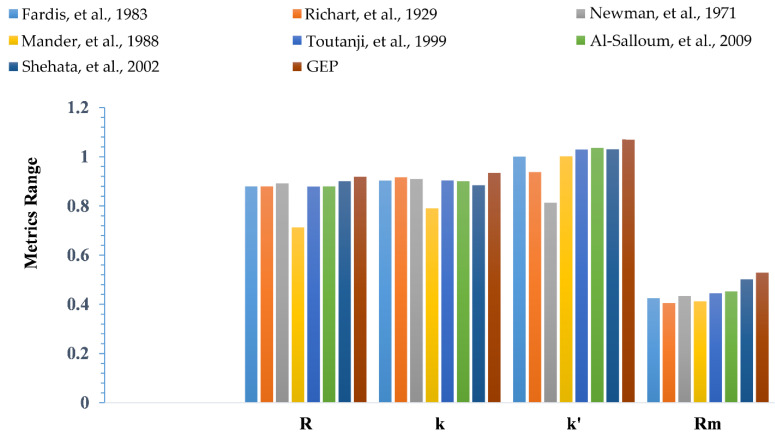
External validation based on the evaluation of proposed and exist-ing models [12,14,15,16,79,84,86].

In addition, as shown in Figure 6, the effects of different requirements acquired by GEP-based modeling for forecasting the *f_cc_* of CFRP confined concrete, indicates the resilience and competence of the generated models in conjunction with other well-known models, since the resulting models fulfill all of the requisite characteristics and dominate other forecasting frameworks significantly. It can be seen that some models did not even fulfill the requirements for external validation, as can be seen in the case of R_m_, which remains less than <0.5 for some models. In addition, it has been observed that merely using R values, or MAE and RMSE values, cannot even comprehend the model’s applicability and accuracy. These metrics significantly contribute towards the generalization and resilience of the model. As presented in the comparative graph, the GEP model provides a significantly improved performance, whereas other models even fail to fulfill some criteria for validation.

### 5.3. Parametric Study

Based on the parametric analysis, the impact of different explanatory parameters on the *f_cc_* for CFRP confined concrete has been assessed in this study; it is evident that *f_cc_* rises in accordance with *f_co_*, *nt*, *h*, and *E*, but it decreases as *d* increases. Parametric analysis is necessary to guarantee that the model outcomes are in agreement with actual outcomes, and to evaluate the resulting model from an engineering perspective. The capacity of analytical expression is to determine how well anticipated values coincide with the underlying computing behavior of the model [49,62,73].

**Figure 7:**
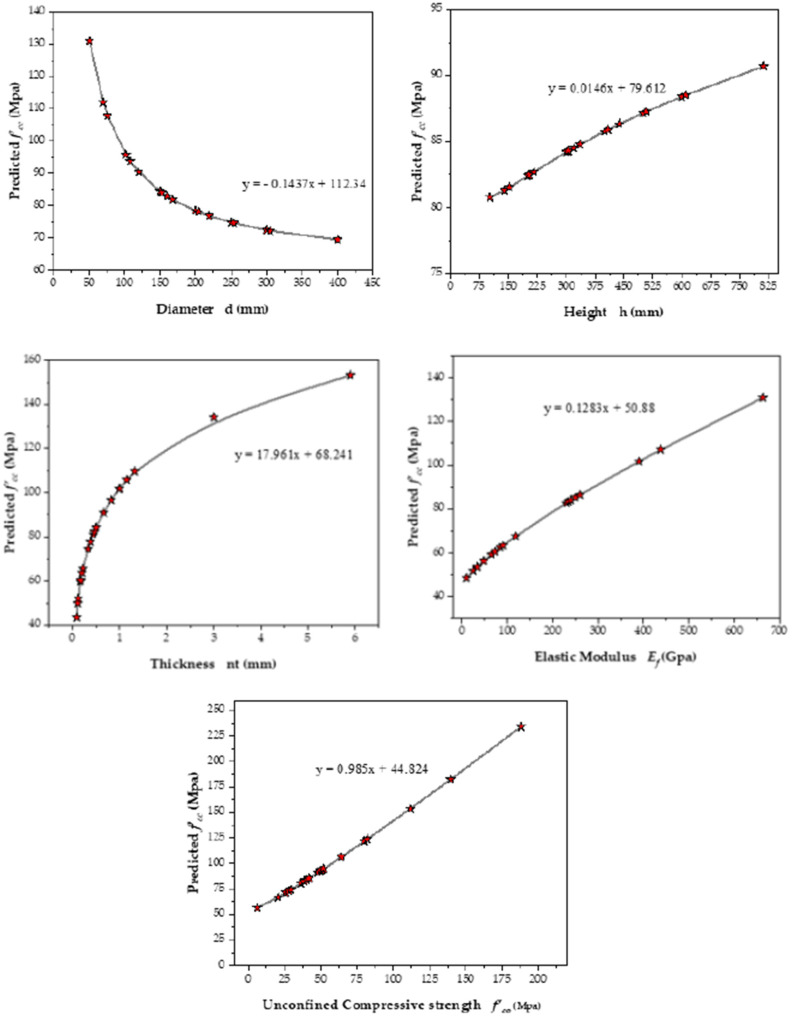
Parametric analysis of input variables.

The parametric analysis depicts the reaction of GEP-based *f_cc_* modeling to respective introductory variables with a certain variability. A predictor variable is changed at a time, whereas the rest of the parameters stay unchanged at the arithmetic mean in the process. Thus, the parametric analysis evaluation is done by creating a set of input data for each parameter based on its distribution in the database. The proposed model is provided with these values, and the *f_cc_* is determined. Each predictor variable’s behavior with regard to the model is then produced by repeating this process using another variable. There are several variables that influence the prediction models perspicuously, such as *d*, *nt*, *E,* and *f_co_*, except for *h*. Figure 7 shows the response of the *f_cc_* prediction models to the changes in these variables. These responses are shown to be in close agreement with the models developed previously in the literature by accurately anticipating the *f_cc_* based on their physical behavior.

### 5.4. Sensitivity Analysis

**Figure 8:**
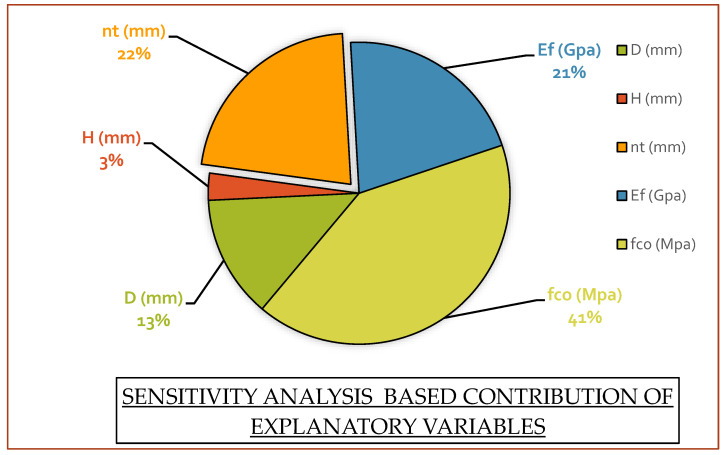
Sensitivity analysis based on the relative contribution of predictor variables.

A sensitivity analysis (SA) is used to determine how a model’s response is impacted by variations in the model’s input parameters. A dependent variable’s response to varying values of predictors can be modeled using the SA method. The SA percentage is derived by utilizing an expression for each input operating parameter [62,63]. The sensitivity index (SI) is calculated by determining the percentage of every variable’s outcome discrepancy. Accordingly, the SI value can be determined in the following manner:

$$\mathrm{SI}=\frac{N_{i}}{\sum_{n}^{j=1}N_{j}}\times 100$$

$$N_{i}=f_{\max}\left(x_{i}\right)-f_{\min}\left(x_{i}\right)$$

where f_max_ and f_min_ are the *i*th input variable’s maxima and minima, other input parameters would remain fixed at respective arithmetic mean, and n represents the number of input parameters. Results from this study’s sensitivity analysis are demonstrated by a pie chart, as shown in Figure 8, for the confinement model of CFRP based confined concrete.

A sensitivity analysis is conducted to evaluate the relative percentage contribution of input parameters to the compressive strength of CFRP confined concrete. The most important parameter is unconfined strength, as it contributes around 41% to the compressive strength of the CFRP confined concrete, whereas around 43% contribution is from confinement alone, and the rest was contributed by geometrical properties such as the diameter and height of the specimens. Based on these percentage contributions it is clear that the selected predictor variables have enough impact on model development, and the same variables could be utilized for future studies. Sensitivity analysis also decides the performance of the developed model on the basis of their contribution, If the variables do not show a considerable contribution, then it would negatively affect the model. As per Figure 8, the GEP framework for *f_cc_* is mostly more responsive to *f_co_*, *nt*, and *E_f_*, than to other variables. These metrics signify that the model’s output is governed by the abovementioned parameters. In addition, the SI values for all other models are based on their dependency on the system parameters. SA is a useful tool for future research, including identifying the most vulnerable input factors for a realistic model’s employment, future trials, and the development of new models; however, it should be noted that each model is distinctive for simulation purposes, irrespective of how accurately it can forecast the target value. Thus, engineers must be intimately conversant with the sensitivity and significance of every parameter in the model they employ for simulation purposes. Thus, the GEP approaches for *f_cc_* are distinguished from other models in that all parameters are effectively implicated in forecasting the outcome, and therefore, the design result.

## 6. Conclusions

This research aimed to see if the GEP methodology could be used to indirectly estimate the confined strength *f_cc_*, for CFRP based confined concrete columns, which are fundamental for the development and construction of concrete structures. As part of an extensive data analysis to date, a new GEP model has been developed. The latest formulation has been presented to forecast *f_cc_*, taking into account the most influential parameters, as per the theoretical and experimental literature. The developed GEP models were evaluated using a variety of statistical measures and other investigations. Considering substantial input variables while modeling the behavior, the model can effectively capture the results that are more accurate and closer to the actual response.

Moreover, professionals must also be well-versed regarding the impacts and effects of input factors on models they employ for development; therefore, an SA comparison has been carried out. The comparison findings based on SA for notable models indicated the distinctiveness of every model’s reaction to individual input parameters. It is important to note that the GEP models developed for the planning and design framework of CFRP confined concrete columns encompass the effects of all impactful explanatory variables, as per the specified independent factors in the literature. The SI values of the GEP models for *f_co_* are 41%, which means that the GEP model is more responsive toward the strength of unconfined concrete than other parameters, which has a significant impact on the design results. Parametric analysis is also considered to analyze the GEP model from an engineering and materials perspective, and the findings were confirmed via laboratory studies in the literature. When it comes to an indirect assessment of the *f_cc_* of enclosed CFRP columns, the presented findings for assessment and validation show that GEP models outperformed previous frameworks in terms of scientific perspectives and performance capability when forecasting the target. In addition to further signifying the accuracy of the GEP model, a comparison is also drawn between the developed regression models and GEP. For this purpose, MNLR model statistics were evaluated in contrast to the developed AI model, and GEP dominates the former one in terms of accuracy statistics. This study shows that the GEP technique can be used as a dependable and robust replacement for conventional procedures for highly nonlinear and complex engineering issues.

## Figures and Tables

**Table 1:** Hyper parameters configuration incorporated for modeling.

Sr. #	Generalized Setting	
1	➢ Chromosome number	120
2	➢ Gene number	3
3	➢ Size of head	8
4	➢ Genes’ linkage function	Addition
5	➢ Set of functions	+, /, −, ×, 3√
	**Constants Configurations**	
6	➢ Constants per gene	10
7	➢ Data type	Floating
8	➢ Bound range	−10 to +10
	**GEP Operators**	
9	➢ Mutation:	0.00138
10	➢ Function Insertion:	0.00206
11	➢ Permutation:	0.00476
12	➢ IS Transposition:	0.00548
13	➢ RIS Transposition:	0.00496
14	➢ Inversion:	0.00548
15	➢ Gene Transposition:	0.00157
16	➢ Random Chromosomes:	0.0026
17	➢ Constant Insertion:	0.00123
	**Recombination Rates**	
18	➢ Uniform	0.00755
19	➢ One-Point	0.00277
20	➢ Two-Point	0.00189
21	➢ Gene	0.00277

**Table 2:** External validation assessment of AI and Regression Models.

Sr. #.	Equation	Range	Model	Output	Reference
1	$R=\frac{\sum_{i-1}^{n}\left(e_{i}-\bar{e}i\right)\left(m_{i-}\bar{m}i\;\right)}{\sqrt{\sum_{i-1}^{n}\left(e_{i}-\bar{e}i\right)²\sum_{i-1}^{n}(m_{i}-\bar{m}i)²}}$	*R > 0.8*	GEP	0.917	
			MLR	0.788	
			MNLR	0.856	
2	$R_{m}=R^{2}\times \left(1-\sqrt{\left|R^{2}-R_{o}^{2}\right|}\right)$	$R_{m}>0.5$	GEP	0.528	(Roy and Roy, 2008) [87]
			MLR	0.244	
			MNLR	0.398	
	where $R_{o}^{2}=1-\frac{\sum_{i=1}^{n}{\left(o-p_{i}^{o}\right)}^{2}}{\sum_{i=1}^{n}{\left(o_{i}-{\underline{\bar{p}}}_{i}^{o}\right)}^{2}},o_{i}^{o}=k\times p_{i}$	$R_{o}^{2}\;≅1$	GEP	0.980	
			MLR	0.987	
			MNLR	0.977	
	$R\prime_{o}^{2}=1-\frac{\sum_{i=1}^{n}{\left(o_{i}-p_{i}^{o}\right)}^{2}}{\sum_{i=1}^{n}{\left(o_{i}-{\underline{\bar{o}}}_{i}^{o}\right)}^{2}},p_{i}^{o}=k\prime \times o_{i}$	$R\prime_{o}^{2}\;≅1$	GEP	0.998	
			MLR	0.997	
			MNLR	0.988	
3	$k=\frac{\sum_{i=1}^{n}\left(o_{i}\times p_{i}\right)}{\sum_{i=1}^{n}o_{i}^{2}}$	0.85<k<1.15	GEP	0.934	(Golbraikh and Tropsha, 2002) [88]
			MLR	0.965	
			MNLR	0.952	
4	$k\prime =\frac{\sum_{i=1}^{n}\left(o_{i}\times p_{i}\right)}{\sum_{i=1}^{n}p_{i}^{2}}$	0.85<k′<1.15	GEP	1.019	
			MLR	0.980	
			MNLR	1.014	

## Data Availability

The data available will be made on request.
